# Update on Novel Non-Operative Treatment for Osteoarthritis: Current Status and Future Trends

**DOI:** 10.3389/fphar.2021.755230

**Published:** 2021-09-16

**Authors:** Tao Chen, Weidong Weng, Yang Liu, Romina H. Aspera-Werz, Andreas K Nüssler, Jianzhong Xu

**Affiliations:** ^1^ Department of Orthopedic Surgery, The First Affiliated Hospital of Zhengzhou University, Zhengzhou, China; ^2^ Department of Trauma and Reconstructive Surgery, BG Trauma Center Tübingen, Siegfried Weller Institute for Trauma Research, Eberhard Karls University Tübingen, Tübingen, Germany; ^3^ Department of Clinical Sciences, Orthopedics, Faculty of Medicine, Lund University, Lund, Sweden

**Keywords:** tissue engineering, osteoarthritis, scaffold, gene, cartilage regeneration, non-operative

## Abstract

Osteoarthritis (OA) is a leading cause of pain and disability which results in a reduced quality of life. Due to the avascular nature of cartilage, damaged cartilage has a finite capacity for healing or regeneration. To date, conservative management, including physical measures and pharmacological therapy are still the principal choices offered for OA patients. Joint arthroplasties or total replacement surgeries are served as the ultimate therapeutic option to rehabilitate the joint function of patients who withstand severe OA. However, these approaches are mainly to relieve the symptoms of OA, instead of decelerating or reversing the progress of cartilage damage. Disease-modifying osteoarthritis drugs (DMOADs) aiming to modify key structures within the OA joints are in development. Tissue engineering is a promising strategy for repairing cartilage, in which cells, genes, and biomaterials are encompassed. Here, we review the current status of preclinical investigations and clinical translations of tissue engineering in the non-operative treatment of OA. Furthermore, this review provides our perspective on the challenges and future directions of tissue engineering in cartilage regeneration.

## Introduction

Osteoarthritis (OA) is a degenerative joint disease and a leading cause of pain and disability among adults (El-Tawil et al., 2016). Over the past decades, along with both an aging population and an increasing obese rate, the incidence and prevalence of OA have a constant growth (Collaborators, 2016). It is estimated to be 18% of females and 9.6% of males aged ≥60 years have symptomatic OA all over the world (Hunter and Bierma-Zeinstra, 2019). The direct medical cost of OA accounts for 1–2.5% of the gross domestic product in high-income countries (March et al., 2014). Nowadays, treatments designed for OA are various. Generally, treatments applied for subjects with mild to moderate OA (Kellgren–Lawrence [K-L] grade 1–3) include education, exercise, weight control, analgesics, and intra-articular (IA) injection of corticosteroids (CSs) or hyaluronic acid (HA) (Ringdahl and Pandit, 2011). These treatments may benefit some patients by reducing pain and improving joint mobility, but none of them can prevent the progressive destruction of cartilage. In advanced disease (K-L grade 4), joint arthroplasty or total replacement surgery has been the ultimate therapeutic option, especially for patients who are unsatisfied with other treatments (Ronn et al., 2011). However, these surgeries are incursive and irreversible procedures and are often accompanied by serious complications (Ronn et al., 2011). In addition, many subjects who suffer from severe OA are relatively young, and they would undergo a second surgery to prevail a useful life. Therefore, the research and development of new therapeutic alternatives are urgent for OA.

On the other hand, OA has been increasingly recognized as a complex syndrome involving the whole joint tissues, while not defined as a single mechanical-induced disorder as before (Chen et al., 2017). Treatments for OA, therefore, have been shifted from the supportive to the preventive or regenerative, aiming at decelerating or reversing the progress of cartilage degeneration (Hunter and Bierma-Zeinstra, 2019). Many attempts have been made to therapeutic procedures for the early treatment of cartilage defects through non-operative approaches. A DMOAD is a drug that prevents the structural demolition of OA coupled with symptom relief (Oo et al., 2018). Some of the DMOADs are being assayed in clinical trials with advanced development (Ghouri and Conaghan, 2019).

In recent years, with the development of life sciences and biomaterials, tissue engineering has become a promising tool for cartilage regeneration (Liu Y. et al., 2017). Tissue engineering is thought a reparative treatment that mainly targets interference at the early stages of OA to maintain and restore the extracellular matrix (ECM) of cartilage (Liu Y. et al., 2017). It offers a possibility to regenerate cartilage by using cells, scaffolds, and genes alone or combined (Morouco et al., 2019).

In this review, we will provide an overview of current and potential non-surgical therapeutic alternatives for OA patients. Representative strategies in preclinical animal models and clinical translations of humans using tissue engineering will be highlighted and discussed.

## Cartilage and OA

Human articular cartilage (AC) is a hyaline connective tissue designed to protect the diarthrodial joints. This highly specialized structure provides the joints with mechanical features, such as load-bearing, low friction, and smooth movement (Archer, 2003). It is comprised of sparsely distributed chondrocytes and a dense ECM, in which cells account for less than 5% of the total mass (Sophia Fox et al., 2009). The primary components of ECM are water, collagen, proteoglycan, and other matrix constituents. In healthy cartilage, water, collagen, and proteoglycan together make up 90–95% of total content, although their proportions vary across the cartilage (Akkiraju and Nohe, 2015).

**FIGURE 1:**
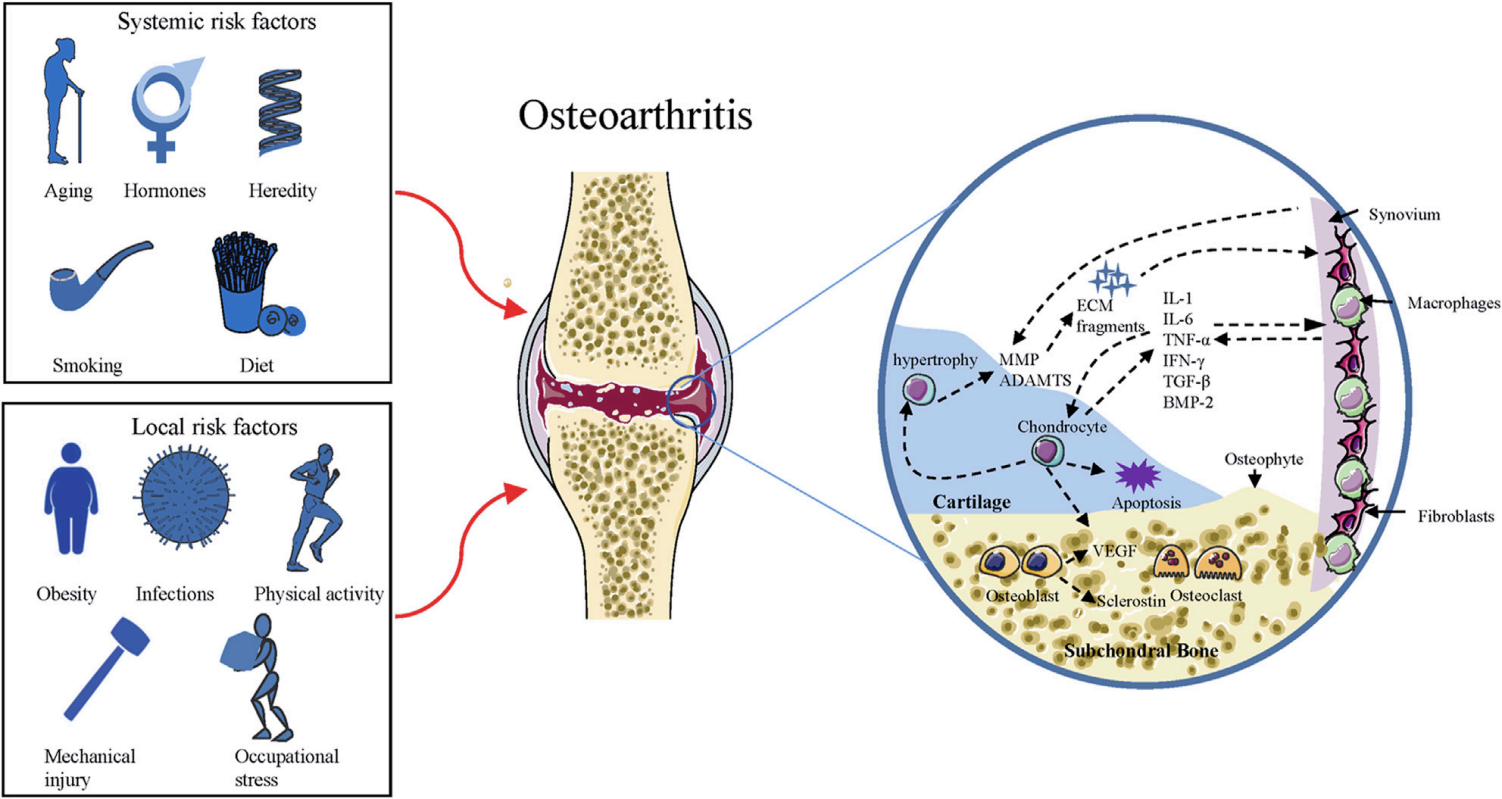
Risk factors and pathogenic process of OA. Squares on the left side include the risk factors responsible for the development of OA, and the circle on the right side represents the pathogenic process of OA. ADAMTS = a disintegrin and metalloproteinase with thrombospondin motifs; MMP = matrix metalloproteinase; ECM = extracellular matrix; IL = interleukin; TNF = tumor necrosis factor; IFN = Interferon; BMP = bone morphogenic protein; TGF = transforming growth factor; VEGF = Vascular endothelial growth factor.

OA is a disease with involvement of the whole joint, characterized by cartilage erosion, subchondral bone remodeling, synovial inflammation, osteophytes formation, as well as degeneration of ligaments and menisci (Chen et al., 2020). It is the most common arthritis associated with several risk factors (as indicated in Figure 1) in the pathogenesis of cartilage degeneration (Silverwood et al., 2015). The disease is an active variation that results from an imbalance between anabolic and catabolic activity, while not a passive degenerative disease or alleged “wear and tear” arthritis as described before (Chen et al., 2017). Although much work has been conducted to understand how the balance is perturbed, it is still not clear-cut. During the process of OA, changes that occur in cartilage are the alteration of cartilage composition and loss of cartilage integrity, which increases its susceptibility to disruption (Goldring and Goldring, 2007). Degenerative shifts in the cartilage lead to increased production of ECM fragments, which promote the release of pro-inflammatory cytokines, like interleukin-1 (IL-1), interleukin-6 (IL-6), and tumor necrosis factor-α (TNF-α) (Scanzello and Goldring, 2012). Once secreted, these cytokines can modulate chondrocytes and adjacent synoviocytes metabolism, inducing the secretion of proteolytic enzymes, such as matrix metalloproteinases (MMPs) and a disintegrin and metalloproteinase with thrombospondin motifs (ADAMTS), which in turn aids in cartilage degradation and fragmentation (Fernandes et al., 2002; Chen et al., 2017). Elevated ECM fragments additionally stimulate the release of pro-inflammatory mediators and matrix degradation products, forming a vicious circle (Fernandes et al., 2002). Parallel to these changes in cartilage, proliferating synoviocytes also release pro-inflammatory and catabolic products, which adversely contribute to ECM degradation (Scanzello and Goldring, 2012). The deviant expression of growth factors, such as transforming growth factor β (TGF-β), bone morphogenic protein 2 (BMP-2), and upregulated immune response, might bring about chondrocyte hypertrophy/apoptosis as well as osteophytes formation (Akkiraju and Nohe, 2015). In OA joints, subchondral bone undergoes remarkable remodeling processes in both composition and structure, including microarchitecture damage, bone marrow lesions, and bone cysts (Hu et al., 2021). Subchondral bone remodeling is an adaptive change to local biomechanical and biological signals, which correspond to Wolff’s Law (Zhu et al., 2020). When the bone is subjected to abnormal load-bearing, a number of bone properties change, including the increased bone mass, subchondral bone thickening, and trabecular restructuring (Zhu et al., 2020). These alterations are mediated by various types of cells, such as osteoblasts, osteoclasts, and osteocytes (Sims and Martin, 2020). Figure 1 summarizes the risk factors and pathogenic process of OA.

Disease severity does not correspond to the level of reported symptoms in OA patients. Some persons endure structural destructions in cartilage while they are asymptomatic (Hunter et al., 2008). Pain is the typical symptom presented in OA and a major driving force for seeking a clinical solution (Hunter et al., 2008). Pain, reduced movement, stiffness, joint instability, and swelling are essential symptoms in diagnosing OA (Hunter and Bierma-Zeinstra, 2019). Radiographic evidence of OA includes narrowing of joint space, subchondral bone thickening, and osteophytes formation (Braun and Gold, 2012).

## Non-Operative Therapies for OA

### Non-Pharmacological and Pharmacological Treatments of OA

At present, there is no cure for OA. Current non-surgical strategies for treating OA contain physical measures and pharmacological therapies. They are normally utilized for patients with mild or moderate OA (K-L grade 1–3) to relieve pain, increase joint motion and improve the quality of life (Ringdahl and Pandit, 2011).

#### Non-Pharmacological Treatments of OA

Nowadays, all guidelines agree that non-pharmacological treatments such as education and self-management, exercise, weight control, and walking aids should be central to the management of patients with OA (Zhang et al., 2010; Nelson et al., 2014). Most of the guidelines recommend strongly that OA patients should acquire up-to-date information and education to allow them to self-manage the disease to a certain extent (Zhang et al., 2008; Block, 2014). Exercise therapy (strengthening exercise and aerobic exercise) is helpful in reducing pain, improving joint motion, and strengthening muscles around the joints (Jordan et al., 2003). Obesity or overweight is associated with the prevalence and progression of knee OA, while weight loss can help to relieve their OA symptoms and delay the structural damage (Messier, 2009). The benefits of knee braces and other assistive devices for physical support and assistance are still controversial and not well-organized (Thomas et al., 2018). Besides, some alternative medicine treatments, like acupuncture, thermal modalities, and therapeutic ultrasound are likely to have little effect in reducing the pain of OA patients (Block, 2014).

#### Pharmacological Treatments of OA

Clinical evidence also suggests that some of OA patients will benefit from drugs (Ringdahl and Pandit, 2011). Drugs, including acetaminophen, non-steroidal anti-inflammatory drugs (NSAIDs), and opioids are essential medicine for patients who have moderate to severe pain. Among these drugs, acetaminophen and NSAIDs are recommended as the first-line pain medication for OA by most guidelines (Dougados, 2006; Hunter and Bierma-Zeinstra, 2019). Nonetheless, safety should be an important consideration in selecting these drugs, since they are reported to be related to considerable side effects, such as liver toxicity, gastrointestinal and cardiovascular complications (Sostres et al., 2010). Opioids are more potent and effective drugs for patients with refractory pain. Both short and long-acting opiates are effective in managing OA pain and have level 3 evidence in their support (Ringdahl and Pandit, 2011). Benefits from the opiates may be acquired, however, frequent adverse effects are associated with these drugs including nausea, dizziness, vomiting, constipation, and sleepiness (Fuggle et al., 2019). In addition, concerns about pharmacologic tolerance, physical dependence suggest the use of opioids should be appropriately dosed and monitored (Lipman, 2001).

There is emerging evidence that IA injections of CSs and HA are helpful for some OA patients (Wernecke et al., 2015; Concoff et al., 2017). CS is known to inhibit the release of inflammatory cytokines in the affected joint and restrain further cartilaginous destruction (Song et al., 2012). IA injections of CSs may provide some patients with temporary symptomatic relief, and a low risk of adverse effects (Wernecke et al., 2015). According to the guideline from Osteoarthritis Research Society International (OARSI), CSs injections should be performed after patients failing or are unsatisfactory with oral analgesic/anti-inflammatory agents, especially for the patients with symptomatic knee OA with effusions or other physical signs of local inflammation (Zhang et al., 2008). HA is a constituent of synovial fluid, while the contents of HA are decreased and compromised in OA joints (Temple-Wong et al., 2016; Chen et al., 2020). Exogenous supplementation of HA is thought to be a visco-supplemental or pharmaceutical therapy for patients with knee OA. They are inferior to CSs in terms of short-term duration, but with likely prolonged symptomatic benefit (Trueba Davalillo et al., 2015). Many complementary medicines (glucosamine sulphate, chondroitin sulphate, ginger, turmeric, etc.) and nutritional supplements have been used to treat OA, but little detail was given and there was no consensus has been achieved (Nelson et al., 2014).

### Potential Drugs for OA

The existing treatments for the management of OA are palliative and often associated with unacceptable side effects. To blunt the epidemic of OA, DMOADs have become a focus of drug development. These products are capable of modifying the structural progression within the joints, as well as ameliorating the symptoms of OA (Ghouri and Conaghan, 2019). These drugs are designed mainly based on the three phenotypes or subpopulations in OA: cartilage, synovial inflammation, and subchondral bone (Oo et al., 2018). DMOADs are largely more targeted than current drugs and can be administered through local injection, which augments the efficacy while diminishing systemic reaction (Hunter and Bierma-Zeinstra, 2019). For example, injectable biologics such as human platelet rich plasma (PRP), Sprifermin (recombinant human fibroblast growth factor 18, rhFGF-18), bone morphogenic protein 7 (BMP-7), or injectable small molecules and drugs such as WNT signaling pathway inhibitors and MMP inhibitors (Fortier et al., 2011; Glynn et al., 2018; Oo et al., 2018; Mobasheri, 2019). There are significant ongoing efforts in this field, and some of the putative DMOADs are in advanced development (phase II or phase III clinical trials) (Hunter and Bierma-Zeinstra, 2019). Currently, no DMOADs have been licensed by regulatory agencies for use but a number of products have shown promising outcomes in clinical trials (see Table 1).

**TABLE 1:** List of ongoing and completed clinical trials on potential DMOADs.

Drug class	Targeted tissue	Mechanism of action	Phase	ClinialTrials. Gov identifier	Status
**FGF-18**	Cartilage regeneration and repair	Stimulating chondrogenesis and ECM through FGF receptor 3	II	NCT01919164	Completed
Sprifermin (AS902330)					
**PRP**		Directing the local MSCs to migrate, divide, and increase collagen and matrix synthesis	II/III	NCT04931719	Completed
Human PRP					
**BMP-7**		Promoting the synthesis of ECM of chondrocytes	II	NCT01111045	Completed
Human recombinant BMP-7					
**Wnt/β-catenin signaling pathway inhibitors**	Cartilage catabolism	Induction of protease production, especially MMPs	II	NCT03122860	Completed
Lorecivivint (SM04690)			III	NCT04520607	Recruiting
**MMPs inhibitors**		Inhibiting the zinc-dependent MMPs	II	NCT00041756	Completed
PG-530,742					
**Senolytics/Senomorphics**		Eliminating or altering senescent cells selectively	I/II	NCT04815902	Recruiting
Fisetin			I/II	NCT04210986	Active, not recruiting
**PTH**	Subchondral bone	Subchondral bone remodeling	II	NCT03072147	Active, not recruiting
Teriparatide					
**MEPE**		Subchondral bone remodeling	II	NCT01925261	Completed
TPX-100					
**Cathepsin K inhibitors**		Inhibiting osteolytic protease by osteoclasts	II	NCT00371670	Completed
Balicatib					
**Calcitonin**		Inhibiting osteoclast bone reabsorption through calcitonin receptor on osteoclasts	III	NCT00486434	Completed
Oral Salmon Calcitonin (SMC021)					
**Anti-IL-1**	Synovial inflammation	Neutralizing IL-1α and IL-1β	II	NCT02087904	Completed
ABT-981					
**Anti-TNF**		Binds specifically to TNF-α and blocks its interaction with endogenous TNF	II	NCT00185562	Completed
Adalimumab					
**iNOS inhibitors**		Inhibiting inducible NO synthase	II	NCT00565812	Completed
Cindunistat hydrochloride maleate (SD-6010)					

FGF = fibroblast growth factor; PRP = platelet rich plasma; BMP = bone morphogenic protein; MMP = matrix metalloproteinase; PTH = parathyroid hormone; MEPE = matrix extracellular phosphoglycoprotein; IL = interleukin; TNF = tumor necrosis factor; ECM = extracellular matrix; iNOS = inducible nitric oxide synthase; NO = nitric oxide.

Sprifermin (rhFGF-18) acts on FGF receptor 3 in cartilage to stimulate chondrogenesis and ECM production *in vivo* and *in vitro* (Reker et al., 2017; Sennett et al., 2018). BMP-7 is a growth factor and has been investigated as a potential drug to repair damaged AC. In addition to the depletion in OA cartilage, BMP-7 also has reparative effects on cartilage by promoting the synthesis of ECM (Thielen et al., 2019). Platelets contain several growth factors, such as insulin-like growth factor-1 (IGF-1), TGF-β, VEGF, as well as chemokines, cytokines, and numerous soluble proteins (Gato-Calvo et al., 2019). The concentration of platelets in the PRP is 4–6 times higher than that of a healthy person’s blood (Qian et al., 2017). It is believed that the clinical efficacy of growth factors could be exerted with PRP, including promoting MSCs recruitment, proliferation, and chondrogenesis (Gato-Calvo et al., 2019). The activation of Wnt/β-catenin signalling pathway can also induce cartilage damage by upregulating the expression of catabolic genes, like ADAMTS and MMPs (Oo et al., 2018). Lorecivivint (SM04690) is a small-molecule Wnt pathway inhibitor and its promising results have been shown in preclinical studies (Deshmukh et al., 2018; Deshmukh et al., 2019). A phase II trial (NCT03122860) conducted on 700 patients for 24 weeks reported a favorable improvement in both pain and function as compared with placebo (Yazici et al., 2021). A small phase-III (NCT04520607) trial is recruiting participants. In addition, senolytics and senomorphics which target pathogenic senescent cells are an emerging therapy for treating aging and chronic diseases (Lagoumtzi and Chondrogianni, 2021). These drugs are also under clinical trials for OA therapy. Fisetin is a polyphenol extracted from fruits and vegetables and has been shown to have senolytic and anti-inflammatory effects (Zheng et al., 2017). Two such clinical trials (NCT04210986, NCT04815902) for Fisetin are underway. Previously, another clinical trial by Unity Biotechnology studying the potential of UBX0101 as a senolytic drug for OA has failed (Hügle and Geurts, 2017). On the other hand, subchondral bone pathologies are indispensable for mediating cartilage damage in OA. Therefore, therapeutic drugs that targeting subchondral bone remodeling is an attractive option for DMOAD development. Teriparatide is a 1–34 amino-acid fragment derived from human parathyroid hormone (PTH) and is normally used as a bone anabolic therapy for osteoporosis. In OA, it displays the ability to stimulate the synthesis of ECM and improve subchondral bone mineral density (Sampson et al., 2011). Matrix extracellular phosphoglycoprotein (MEPE) is a protein that regulates bone and growth plate cartilage mineralization, while TPX-100 is a novel 23-amino-acid peptide that is derived from MEPE (Oo et al., 2018). At the 2020 OARSI conference, it was reported that a significant reduction in pathologic bone shape changes of the femur after IA injection of TPX-100 at 12 months (McGuire et al., 2020). Cathepsin K is a potent osteolytic protease for bone resorption, and cathepsin K inhibitor has been reported to attenuate cartilage damage in animal models of OA (Hayami et al., 2012). Calcitonin is a hormone secreted by the parafollicular thyroid cells and inhibits osteoclast activity in bone through affecting the calcitonin receptor localized to osteoclasts. Several animal studies have been demonstrated that salmon calcitonin has positive effects on disrupt cartilage degeneration. In a 2-years phase III trial (NCT00486434), oral calcitonin significantly improved knee function in OA patients and enhanced cartilage thickness compared to placebo. IL-1 and TNF-α are the most extensively studied cytokines in preclinical studies. However, most clinical trials investigating the inhibitor of IL-1 and TNF-α failed to meet symptomatic benefits in OA patients (Oo et al., 2018). Nitric oxide (NO) is an inflammatory mediator and is produced by the inducible NO synthase (iNOS) pathway. Cindunistat hydrochloride maleate (SD-6010) is an orally administered inhibitor of human iNOS, while it failed to show the rate of change in joint space narrowing (JSN) in a 2-years phase II trial (NCT00565812) (Hellio le Graverand et al., 2013).

## Non-Surgical Application of Cartilage Tissue Engineering

Owing to the avascular nature of cartilage, damaged cartilage has limited capability for healing and repairing by itself (Chen et al., 2017). Therapies, like non- or pharmacologic treatment, only focus on relieving the symptoms of OA, instead of regenerating damaged or degenerative cartilage. These conditions, thus contributing to the emergence of cartilage tissue engineering (CTE), which is come up as a promising strategy for cartilage regeneration by integrating methods and perspectives from life sciences and biomaterials (Ondresik et al., 2017). The goal of CTE is to modify the host microenvironment of OA and promote cartilage regeneration (Ondresik et al., 2017). Successful tissue engineering depends on multiple aspects, including appropriate cells for implantation, well-designed scaffolds for mechanical support, and biological factors for directing cells to differentiate in the proper direction (Liu Y. et al., 2017; Morouco et al., 2019). Recent investigations have highlighted its promising, and some strategies are already available on the market (Brittberg, 2008; Evans et al., 2018).

### Cell Injection Therapy

When it comes to cell therapy, it normally refers to chondrocytes or mesenchymal stem cells (MSCs) based regimen (Phull et al., 2016; Lee and Wang, 2017). Considering the demerits of complicated operation and donor site mobility in chondrocytes-based treatment, MSCs are more prone to be accepted as a fitting source of cells in cell-based therapies (Im, 2018). MSCs are cells of mesodermal origin and progenitors of various cells (osteocytes, chondrocytes, adipocytes, etc.), and can be isolated from diverse tissues, including bone marrow, adipose tissue, placenta, and even peripheral blood (Nejadnik et al., 2015). MSCs have a higher proliferation rate, chondrogenic differentiation capacity and immunomodulatory abilities, which are key features in cartilage regeneration (Gupta et al., 2012). Additionally, the anti-inflammatory and immunosuppressive attributes of MSCs imply that they might inhibit synovial inflammation and reduce pain, as well as can be used as both autografts and allografts for OA patients in clinical trials (Im, 2018).

#### MSC-Based Therapy for IA Injection of OA

The efficacy of MSC-based therapy has been proven to be effective in different OA animal models (Gupta et al., 2012; Grassel and Lorenz, 2014; Sasaki et al., 2019), ranging from murine, rabbit, canine, ovine, and caprine to equine (see Table 2). *In vivo* study, IA injection of human adipose tissue-derived MSCs (AD-MSCs) engrafted into the rat joints and increased the cartilage thickness in surgery-induced OA animal models (Li et al., 2016). Small animal models are often used as a proof-of-concept, owing to their AC is thinner and smaller than humans. Large animals, such as ovine, equine, or sheep are more suitable for modeling human AC defects and investigating the effectiveness of MSC-based treatments (Grassel and Lorenz, 2014). In a study conducted by Al Faqehand colleagues, autologous bone marrow MSCs (BM-MSCs) were performed for IA injection of surgically-induced OA sheep. 6 weeks after injection, gross evidence of retardation of cartilage destruction was observed in the OA joints treated with BM-MSCs (Al Faqeh et al., 2012). Consistent with this study, the study of Ko *et al.* found that injected SOX-6, 9-transfected AD-MSCs attenuated the progression of OA in goats (Ko et al., 2019). The safety and feasibility of IA injection of MSCs for cartilage repair have also been evaluated in several clinical trials (Grassel and Lorenz, 2014; Lee and Wang, 2017). It is suggested that subjects with mild to moderate OA (K-L grade 1–3) are optimal candidates for MSC therapy (Lee and Wang, 2017). Jo *et al.* enrolled 18 patients with knee OA (K-L grade 2–3) and injected autologous AD-MSCs into the knee, showing improved Western Ontario and McMaster Universities Osteoarthritis Index (WOMAC) score, decreased size of the cartilage defect, and increased cartilage volume at 6 months after injection in the high-dose group (1.0 × 10^8^/L) (Jo et al., 2014). Concomitantly, in a 2 years’ follow-up study, Orozco et al. found that IA injection of autologous expanded BM-MSCs was effective in improving both magnetic resonance imaging (MRI) T2 mapping and visual analogue scale (VAS) outcomes in patients undergoing chronic knee pain (Orozco et al., 2013; 2014). In addition, one randomized controlled trial (RCT) study assessing the feasibility and safety of treating OA with allogeneic BM-MSCs was reported by (Vega et al. (2015) *.* In this study, 30 patients with chronic knee OA were randomized into 2 groups of 15 patients: the test group received allogeneic BM-MSCs (40 × 10^6^ cells) by IA injection, while the control group received HA (60 mg). After 12 months, the test group illustrated significant improvement in algofunctional indices and quantification of cartilage quality compared to the control group (Vega et al., 2015). Nevertheless, patients withstanding larger cartilage lesions exhibited significantly inferior consequences (Im, 2018). Additionally, MSCs obtained from subjects with severe OA have decreased potential for proliferation and chondrogenic differentiation, resulting in a lower cell yield and higher osteogenic differentiation (Murphy et al., 2002).

**TABLE 2:** Animal models using IA injection of MSCs for treating OA.

Category	Cell source	Dose/graft type	Combination use	Model	Evaluation method	References
Small animal study	BM-MSCs	1*10^5^cells/allogeneic	—	mouse	Histology, μCT	Diekman et al. (2013)
	AD-MSCs	2*10^4^ or 2*10^5^ cells/xenogeneic (equine)	—	mouse	Histology	Maumus et al. (2016)
	SM-MSCs	1*10^6^ cells/allogeneic	—	mouse	Clinical score, histology	Yan et al. (2017)
	BM-MSCs	6*10^5^ or 1.3*10^6^ cells/xenogeneic (human)	HA	rat	Gross morphology, histology, pain response	Gupta et al. (2016)
	AD-MSCs	2.5*10^6^cells/xenogeneic (human)	—	rat	Histology, IHC	Li et al. (2016)
	SM-MSCs	1*10^6^ cells/xenogeneic (human)	—	rat	Gross morphology, histology, IHC	Ozeki et al. (2016)
	UC-MSCs	1*10^5^ or 1*10^6^ cells/xenogeneic (human)	Microcryogel	rat	Gross morphology, histology, μCT	Xing et al. (2020)
	BM-MSCs	1*10^6^ cells/allogeneic	HA	rabbit	Gross morphology, histology, IHC	Chiang et al. (2016)
	AD-MSCs	2*10^6^ cells/autologous	HA	rabbit	Gross morphology, histology, IHC	Kuroda et al. (2015)
	SM-MSCs	5*10^6^ cells/autologous	—	rabbit	Gross morphology, histology	Jia et al. (2018)
Large animal study	BM-MSCs	7*10^6^ cells/xenogeneic (human)	HA	pig	Gross morphology, histology	Sato et al. (2012)
	BM-MSCs	1*10^7^ cells/allogeneic	HA	dog	Gross appearance, MRI, histology, IHC	Li et al. (2018)
	AD-MSCs	1*10^7^ or 5*10^7^ cells/allogeneic	HA	sheep	Gross morphology, histology, MRI, μCT	Feng et al. (2018)
	AD-MSCs	1.8*10^6^, 6*10^6^ or 1.8* 10^7^ cells/xenogeneic (human)	—	goat	Histology, macroscopic and micro-scopic scores	Ko et al. (2019)
	BM-MSCs	1*10^7^ cells/autologous	—	horse	Clinical and radiographic scores	Magri et al. (2019)

BM-MSCs = bone marrow mesenchymal stem cells; AD-MSCs = adipose-derived mesenchymal stem cells; SM-MSCs = synovial membrane-derived mesenchymal stem cells; UC-MSCs = umbilical cord-derived mesenchymal stem cells; IHC = immunohistochemistry; HA = hyaluronic acid; MRI = magnetic resonance imaging; μCT = micro-computed tomography.

#### Bottleneck of Cell Injection Therapy for OA

Although encouraging symptomatic relief has been demonstrated in clinical reports, hyaline AC regeneration was rarely reported. In a study with three case reports, Wakitani et al. found that the cartilage defect had been repaired with the fibrocartilaginous tissue in the first patient after 1 year of BM-MSCs transplantation. MRI results of the second patient revealed complete coverage of the defect, while not able to determine the covered materials was hyaline cartilage (Wakitani et al., 2007). Discoveries from animal researches implicated that MSCs achieve therapeutic effects in virtue of paracrine mechanisms, rather than integrating with cartilage directly and producing ECM *in situ* (Saulnier et al., 2015; Ozeki et al., 2016). These paracrine factors consist of various proteins and could be conveyed in extracellular vehicles (EVs) (Lee and Wang, 2017). There are three major categories of EVs: apoptotic bodies, exosomes or nanovesicles, and microparticles/microvesicles (MPs) (Mianehsaz et al., 2019). Recently, it has been increasingly supported that MSCs-derived exosomes contribute to the reparative effects of MSC-based treatment in OA models (Cosenza et al., 2017) (Zhang et al., 2019). An *in vivo* study revealed that BM-MSCs and MSCs-derived exosomes equally protected collagenase-induced mice from joint damage (Cosenza et al., 2017). Other issues such as frequency of injection, the dose of MSCs, MSC origin, and transplantation type also should be taken into consideration (Im, 2018). Moreover, the safety of IA injection of MSCs is one of the key points in clinical application. In one meta-analysis, Peeters assessed the reported adverse events of IA treatment with cultured stem cells in humans, four serious adverse effect cases were reported, including one infection, one pulmonary embolism, and two tumors (Peeters et al., 2013). Lastly, a lacking of a large number of multicenter data and high-level evidence hinders the application of MSCs in early-stage OA. Given current knowledge, the preliminary results indicated that IA MSCs injection is promising in reducing pain and improving the quality of OA patients. However, more RCTs and high-level evidence are still required before in-depth clinical translation.

### Injectable Hydrogel

Various natural or synthetic materials have been explored as scaffolds in CTE to create three-dimensional (3D) tissue constructs that maintain and restore the function and structure of the cartilage. In the application of cartilage regeneration, injectable hydrogels over the solid scaffolds initiate the possibility of percutaneous injection, owing to their highly aqueous 3D cross-linked network structure (Wu et al., 2020). Hydrogels are mainly composed of natural biomaterials (chitosan, fibrin, alginate, collagen, and silk), or synthetic materials (polyethylene glycol (PEG), polyvinyl alcohol (PVA), polylactic acid (PLA)) (Liu M. et al., 2017; Li et al., 2019). Their high porosity allows encapsulated cells to adhere, diffuse, and functionally differentiate within the materials. One of the unique superiorities of hydrogels is that they have similar properties to native ECM of cartilage, providing a beneficial growth environment for chondrocytes to maintain their phenotype (Wu et al., 2020). In addition, injectable hydrogels have the ability to gel *in situ* through controlling some factors such as PH and temperature (Singh et al., 2018). Therefore, it is widely accepted that hydrogel is a potential promising choice for cartilage repair.

#### Preclinical Studies

Notably, over the past decade, the cartilage regeneration potential of hydrogels combined with or without cells and biologics have been investigated and remarkable successes have been achieved in fundamental studies (Roberts et al., 2011; Kontturi et al., 2014; Arora et al., 2017). Kontturi et al. designed an injectable, *in situ* forming type II collagen/HA hydrogel and found that it could assist the long-term survival of the encapsulated chondrocytes as well as maintain their round shape *in vitro* (Kontturi et al., 2014). In another study, Roberts et al. demonstrated that a chondrocyte-laden PEG-LA hydrogel (consisting of PEG and oligomers of lactic acid (LA)) significantly improved the formation of cartilage-like tissue comprised of glycosaminoglycan and collagen under loading (Roberts et al., 2011). Moreover, Arora et al. have reported that TGF-β1 loaded hydrogels enhanced cell survival and chondrogenesis of MSCs and chondrocytes (Arora et al., 2017). In parallel, many preclinical studies have assessed the reparative effects of injectable hydrogels on the cartilage in large and small animal models (Na et al., 2007; Wang et al., 2007; Vilela et al., 2015). Na et al. developed a thermo-reversible hydrogel construct blended with HA, which was used as an injectable carrier for rabbit chondrocytes and TGF-β3. The results showed that when the blended hydrogels mixed with TGF-β3 and chondrocytes were injected subcutaneously into the nude mice, the level of cartilage associated ECM production was significantly higher than those without HA or TGF-β3 (Na et al., 2007). Wang et al. used a chondroitin sulphate (CS) based adhesive-hydrogel to stimulate cartilage formation in goat models. This study suggested greater defects fill in the presence of the hydrogels compared to microfracture alone (Wang et al., 2007).

#### Clinical Trials

Clinical trials of injectable hydrogels on OA treatment are very limited, and their applications are mainly to boost the efficacy of existing therapies, such as microfracture, osteochondral grafting (Wu et al., 2020). Most of the reported trials are case reports or case series. In a pilot clinical study, Sharma *et al.* recruited 18 subjects with focal cartilage defects on the medial femoral condyle. 15 subjects (treated group) were treated with the adhesive-hydrogel in conjunction with microfracture, while three subjects (control group) were treated with microfracture alone. At 6 months follow up, MRI analysis showed significant improvement of repair tissue integration was achieved in the treated group. The treated group also experienced pain reduction compared to the controls, and no major adverse events were observed (Sharma et al., 2013). Another clinical trial using ChonDux hydrogel (consisting of a PEG/HA network and a CS adhesive) for treating full-thickness femoral condyle defects (Wolf et al., 2020). Researchers found that ChonDux mediated 94.2 ± 16.3% of final defects fill over 2-years of follow-up and the treated tissue was similar to adjacent cartilage between 12 and 24 months, suggesting ChonDux is a safe accessory to microfracture therapy (Wolf et al., 2020). In addition, a novel medicine product (Cartistem), comprised of HA hydrogel and allogeneic human umbilical cord blood-derived MSCs (hUCB-MSCs) was assessed in a clinical study over 7-years of follow-up. The results suggested this product appears to be safe and effective for cartilage regeneration in knee OA (Park et al., 2017). The above pilot studies confirmed the prospects of injectable hydrogel in the treatment of OA. However, some challenges for clinical translation remain to be addressed, including the source of the material, and integration to the cartilage in a stable way. Future studies are needed to address these issues before clinical application.

### Gene-Based Therapy

As a disease with a great degree of heritability, genetic changes in OA could contribute to defects of a structural component, or imbalance in the metabolism of cartilage and bone (Mobasheri, 2019). In this regard, gene therapy represents an innovative approach to the medical management of OA. It was firstly reported in articulations by Evans et al, who utilized an anti-arthritic cytokine (IL-1 Receptor Antagonist, IL-1Ra) gene in human joints with rheumatoid arthritis (RA) (Evans et al., 1996). Unlike the protein-based treatments which have a short half-life, gene therapies aim to establish persistent, endogenous synthesis of the *trans*-gene products at target sites through IA injection (Mobasheri, 2019). Recently, this has been carried out *in vivo* and *ex vivo* studies, using different delivery vectors (non-viral or viral), target genes (growth factors, transcription factors, anti-inflammatory cytokines, cell signaling protein iHH, ECM protein, integrin-β1), and cells (stem cells, chondrocytes) with or without biomaterials (Heiligenstein et al., 2011; Shui et al., 2013).

#### Gene Delivery Strategies

Delivery of the transgene can be achieved by viral or non-viral methods. At present, viral vectors are more preferred and have been used for IA gene delivery in animal models (Kay et al., 2009; Wang et al., 2016) and human trials (Mease et al., 2009; Mease et al., 2010). Retroviruses and adeno-associated virus (AVV) are the only vectors that have been assayed in clinical studies until now. Compared with other viral vectors, AAV has the advantage of penetrating deeply within cartilage to transduce chondrocytes *in situ* (Madry et al., 2003)*. In vivo* experiments by Kay et al. showed that the level of IL-1Ra expression present in the joint space of self-complementary AAV (sc-AAV) injected rabbits for 2 weeks in sufficient quantities to suppress inflammation of the IL-1β-induced arthritis model (Kay et al., 2009). Similarly, in another study, Wang et al. suggested that no local or systemic toxicity was found following sc-AAV vector carrying IL-1Ra transgene (sc-rAAV2.5IL-1Ra) injection in mono-iodoacetate (MIA)-induced OA rats (Wang et al., 2016). Sustained expression of IL-1Ra and a low rate of cartilage loss were observed in the vector-injected knees (Wang et al., 2016). In large mammalian joints, transgenic expression of human IL-1Ra could last for 10 weeks at biologically relevant levels following delivery with recombinant AAV in the forelimb joints of horses (Watson et al., 2013). In a phase I clinical trial (NCT00617032), 15 subjects (aged ≥18 years) with inflammatory arthritis (14 with RA and 1 with ankylosing spondylitis) received a single IA injection of rAAV-2 containing a TNF immunoglobulin Fc fusion gene (rAAV2-TNFR:Fc). The result showed that rAAV2-TNFR:Fc appears to be safe and well tolerated in patients not systemically taking TNF-α antagonists (Mease et al., 2009). Furthermore, a phase I/II clinical trial (NCT00126724) on 127 patients (aged 18–75 years) demonstrated that greater improvement in patients treated with rAAV2-TNFR:Fc compared to placebo patients (Mease et al., 2010). Meanwhile, another phase I study (NCT02790723) using AAV is still in progress and will evaluate the effects of IL-1Ra expression on the OA phenotype (Bellavia et al., 2018).

#### Selection of Transgene

Among the numerous target genes, the expressions of GFs and anti-inflammatory cytokines are of interest to researchers and are being assessed in clinical trials (see Table 3). As described aforementioned, IL-1 and TNF-α receptor antagonists are the candidate transgenes with an anti-inflammatory action that have been applied in clinical trials (Mease et al., 2009; Bellavia et al., 2018). TGF-β1 is a growth factor that can augment the ability of chondrogenesis of MSCs. Several preliminary studies have confirmed the effects of TGF-β1 in the repair of cartilage defects and impeding the chondrocytes hypertrophy (van Caam et al., 2015; Xie et al., 2016). TGF-β1 is also the exclusive gene currently being tested in clinical trials for OA treatment (Ondresik et al., 2017). Noh et al. conducted a pre-clinical study to evaluate the efficacy, biodistribution, and safety of single IA injection of the cell mixture (3:1 ratio of genetically unmodified and TGF-β1-secreting human chondrocytes, TG-C) in SCID mice, rabbits, and goats with damaged joints. The results showed that the mixture was tolerated well in all of the species, and cartilage regeneration was present in defects of rabbits and goats (Noh et al., 2010). After these positive pre-clinical results, a phase I clinical trial (NCT00599248) performed on 12 subjects with advanced knee OA suggested that TG-C contributed to an improvement of OA symptoms as well as minor injection site reactions (Ha et al., 2012). Concomitantly, a phase II clinical study carried out on 102 patients (NCT01221441) with knee OA, indicated that TG-C treated patients had positive effects on function elevation and pain mitigation compared to placebo at 1-year follow-up (Cherian et al., 2015). Notably, an *ex vivo* TGF-β1 gene therapy was authorized in Korea for IA injection of knee joints with moderate-to-severe OA. The product (Invossa™) has received marketing approval in Korea and a phase III clinical trial is expected to begin shortly in the United States (Evans et al., 2018). Other genes, like IGF-1, BMPs, cell signaling protein iHH, ECM component (COMP), and integrin β1, are still in their infancy (Bellavia et al., 2018; Tendulkar et al., 2019). Gene combinations are also of interest, and co-infection of IL-1Ra and IGF-1 has a positive effect on repairing cartilage defects *in vivo* (Morisset et al., 2007).

**TABLE 3:** Registered clinical trials on the gene therapy of OA.

Gene	Method of delivery	Number of participants	Evaluations	Phase	ClinialTrials.gov identifier	Status
TGF-β1	Retrovirus, *ex vivo*	12	Safety and biological activity of TissueGene-C in degenerative arthritis patients	I	NCT00599248	Completed
TGF-β1	Retrovirus, *ex vivo*	12	Efficacy and safety of TissueGene-C in degenerative arthritis patients	I	NCT02341391	Completed
TGF-β1	Retrovirus, *ex vivo*	102	Efficacy and safety of TissueGene-C in Patients with grade 3 chronic degenerative joint disease of the knee	II	NCT01221441	Completed
TGF-β1	Retrovirus, *ex vivo*	28	Efficacy and safety of TissueGene-C in degenerative arthritis patients	II	NCT02341378	Completed
TGF-β1	Retrovirus, *ex vivo*	18	Efficacy and safety of TissueGene-C mixed with Fibrin-glue in patients with degenerative arthritis	II	NCT01825811	Completed
TGF-β1	Retrovirus, *ex vivo*	54	Efficacy and safety of TissueGene-C in degenerative arthritis patients	II	NCT01671072	Completed
TGF-β1	Retrovirus, *ex vivo*	163	Efficacy and safety of TissueGene-C in degenerative arthritis patients	III	NCT02072070	Completed
TGF-β1	Retrovirus, *ex vivo*	510	Safety and efficacy of TissueGene-C in patients with grade 2–3 knee OA	III	NCT03203330	Active, not recruiting
IL-1Ra	AAV, *in vivo*	9	Safety of IA Sc-rAAV2.5IL-1Ra in patients with moderate OA of the Knee	I	NCT02790723	Recruiting

Gene therapy offers a novel approach to address the issue of transferring exogenous pharmaceuticals into joints topically and durably. Nevertheless, safety and effectiveness are still hurdles for clinical application. Some authors expressed their concerns about the safety of viral vectors, in particular, after the occurrence of severe adverse events such as leukemia and death (Frank et al., 2009). Although these events were not correlated with viral vectors, the proper monitoring for further clinical application needed to be highlighted.

## Summary and Future Outlook

Recognition of OA is a complex disease with multifactorial nature, and the whole joints are involved in the degenerative process is crucial for cartilage repair. Therefore, it is necessary to increase our knowledge in basic sciences to comprehensively understand the mechanism of different joint components in OA pathology. Until now, in the clinics, conservative management, including physical measures and pharmacological therapy are still the first choices offered for OA patients. Joint arthroplasties or total replacement surgeries are served as the ultimate therapeutic option to rehabilitate the joint function of patients who withstand severe OA. However, these approaches are not able to induce healing processes or halt the degenerative processes in the joints. Demand for cartilage regeneration remains a big challenge both for clinicians and researchers.

Thanks to the innovations and advances in biomaterials and biotechnology, more and more research efforts have been devoted to studying cartilage repair through non-surgical approaches. Stem cell therapies and injectable hydrogels targeting articular cartilage are being largely explored (Lee and Wang, 2017; Yang et al., 2017). Human clinical trials using IA injection of MSCs have taken place, with more and more pending trials listed on Clinicaltrials.gov (Lee and Wang, 2017). The main problem of cell-based therapy is the chondrogenesis of stem cells is often followed by osteogenesis and hypertrophy. Moreover, increasing evidence demonstrates that the number and life expectancy of injected MSCs *in situ* is much lower than expected (Sasaki et al., 2019). Significant efforts have been made to address these issues. Excitingly, it was found that the presence of anti-angiogenic factors, such as gremlin-1, chondromoduli-1, and PTH-related protein was able to suppress chondrocyte hypertrophy and enhance MSC chondrogenesis (Vortkamp et al., 1996; Shukunami et al., 2005; Nagai et al., 2010). Recently, advances in the development of cell-laden hydrogels have opened up new possibilities for cell therapy. Cells in hydrogels adhere to and extend on a 3D environment, which is similar to the morphology and distribution of cells in native cartilage (Wu et al., 2020). In addition, hydrogels are usually used as controlled release carriers for bioactive molecules or target drugs. Bioactive molecules, such as growth factors and cytokines, play essential roles in the metabolism and differentiation of chondrocytes (Fortier et al., 2011; Wojdasiewicz et al., 2014). However, it is challenging to maintain the effectiveness of the medicines encapsulated in the hydrogels. In this regard, Spiller et al. have recently developed a hybrid scaffold consisting of degradable poly (lactic-co-glycolic acid) (PLGA) microparticles and PVA hydrogel in which IGF-1 was loaded, resulting in the release of IGF-1 in a sustained manner over 6 weeks (Spiller et al., 2012). Another challenge for injectable hydrogel is how to firmly integrate the hydrogels with local structures, in particular, utilizing an injectable approach. The emergence of gene transfer provides a novel way to solve some of the widespread, demanding, and intractable problems of modern medicine. Theoretically, gene therapy permits longer-lasting, targeted, location-specific expression of a protein of interest, in a more physiologically relevant way. Preclinical studies have confirmed the efficacy and safety of gene therapy and implicated its prospects. Progress towards clinical application appears to be distant, due to the concerns about viral vectors. It is noteworthy that the first gene product, Invossa™, has received a license in Korea in 2017 (Evans et al., 2018). Its approval will arouse interest in this field to accelerate the development of genetic therapeutics for defective joints.

**FIGURE 2:**
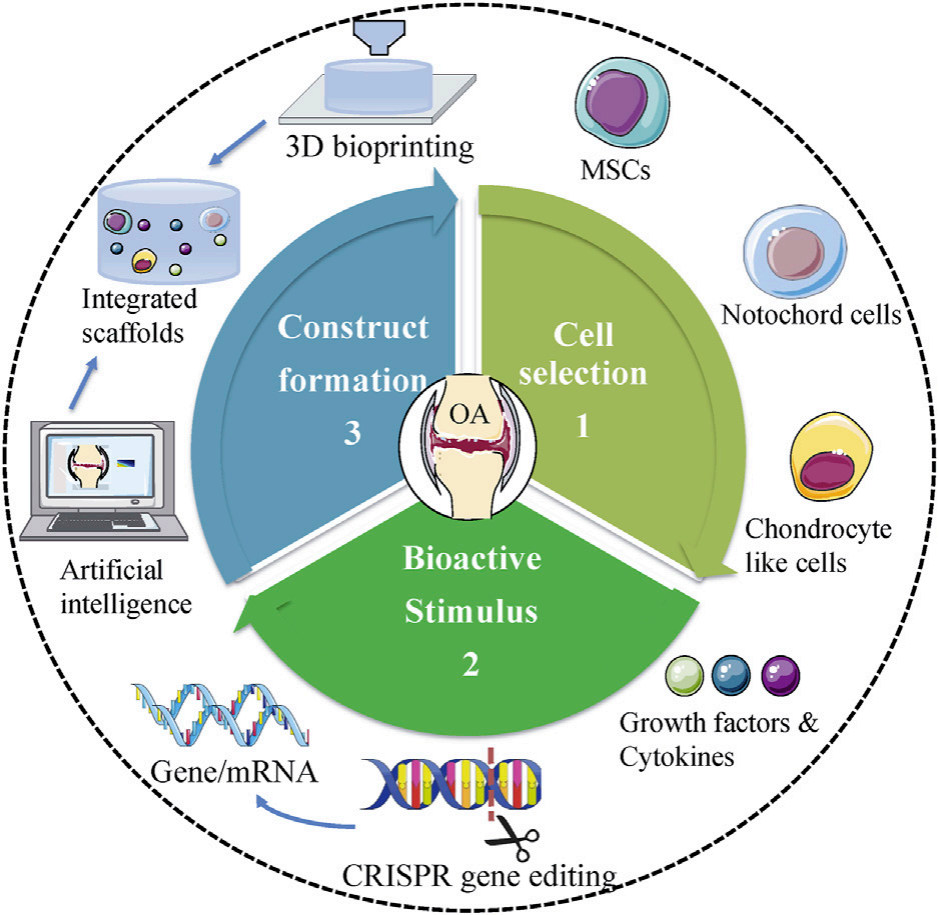
Future trends of tissue engineering in treating OA. Proper cell selection, appropriate bioactive stimulus, and perfect design of scaffolds are central to tissue engineering. Novel scientific technologies, including CRISPR gene editing, 3D bioprinting, and AI should be used to facilitate the development of tissue engineering in treating OA.

Overall, each type of treatment has its merits and demerits concerning the application. To date, no technique has indicated the ability to generate native cartilage in the joints. Therefore, to design more efficient therapies that minimize adverse consequences, it is mandatory to implement interdisciplinary and translational studies. Tissue engineering should be directed to identify the interactions between cells, scaffolds, and the microenvironment of the implant. Recently, Madry et al. designed an injectable and thermosensitive hydrogel on basis of poly (ethylene oxide) (PEO)–poly (propylene oxide) (PPO)–PEO poloxamers, capable of controlling the release of a therapeutic (SOX9) rAAV vector in a clinically relevant minipig model with full-thickness chondral defects (Madry et al., 2020). Four weeks postoperatively, multiple standardized analyses (integration, morphology, matrix staining, and histological scoring of cartilage repair) indicated SOX9/hydrogel construct significantly improved cartilage repair. In addition, the absence of immune cells in all the defects confirmed the benefits of utilizing biomaterial-guided carriers for gene delivery in the clinical application (Madry et al., 2020). Novel scientific technologies would also aid in the development of tissue engineering (Figure 2). CRISPR/Cas9 system is an advanced genome-editing technique that is able to make gene deletion, correction, and substitution, or other changes at specific sites of the genome (Tanikella et al., 2020). For example, CRISPR/Cas9 has been employed to regulate MMP-13 protein levels and enzymatic activity in human chondrocytes. The results showed CRISPR/Cas9 mediated genome editing significantly reduced the level of MMP-13 protein and enhanced collagen II accumulation (Seidl et al., 2019). On the other hand, advanced manufacturing techniques, such as microfluidic biofabrication, 3D bioprinting are required to fabricate complex tissue constructs (Lopa et al., 2018; Roseti et al., 2018). For example, in order to mimic the nature of AC, Stichler’s group utilized a thiol-functionalized HA (HA-SH) hybrid hydrogel embedded with human and equine MSCs as bioink for 3D bioprinting. Embedded MSCs showed a good survival for at least 21 days *in vitro* culture. Concomitantly, double printing with thermoplastic poly (ε-caprolactone) (PCL) made the constructs more mechanically stable and robust (Stichler et al., 2017). Furthermore, with the development of machine learning and artificial intelligence (AI), we may be able to accelerate the process of trial and design better materials for cartilage regeneration in the future (Liu et al., 2020).

In conclusion, there is no doubt that tissue engineering has the potential to reproduce the cartilage through a non-operative approach. Despite the relatively successful preclinical investigations and advanced development of clinical trials have been achieved, several hurdles still exist in the routine to the final clinical practice. In particular, ethical concerns and safety issues regarding cell and gene delivery. Another challenge is the precise design of hydrogels with good biocompatibility, excellent biodegradability, and proper mechanical features. Lastly, it is challenging to integrate the hydrogels with the adjacent cartilage tissues in a stable way. Notably, some tissue engineering-based products, like Invossa™, ChonDux, Cartistem have received marketing approval. It is desirable that these approvals will provoke research interests in this field and therefore accelerate clinical translations in the future.
